# *In vitro* effects of the new oral β-lactamase inhibitor xeruborbactam in combination with oral β-lactams against clinical *Mycobacterium abscessus* isolates

**DOI:** 10.1128/spectrum.00084-24

**Published:** 2024-06-06

**Authors:** Izumi Yamatani, Akio Aono, Keiji Fujiwara, Takahiro Asami, Keisuke Kamada, Yuta Morishige, Yuriko Igarashi, Kinuyo Chikamatsu, Yoshiro Murase, Hiroyuki Yamada, Akiko Takaki, Kosaku Komiya, Satoshi Mitarai

**Affiliations:** 1 Department of Mycobacterium Reference and Research, Research Institute of Tuberculosis, Japan Anti-Tuberculosis Association, Tokyo, Japan; 2 Respiratory Medicine and Infectious Diseases, Oita University Faculty of Medicine, Oita, Japan; 3 Department of Basic Mycobacteriosis, Nagasaki University Graduate School of Biomedical Sciences, Nagasaki, Japan; CNRS-University of Toulouse, Cedex, France; Karolinska Institutet, Stockholm, Sweden; APHP, Hôpitaux universitaires Lariboisière-Saint Louis, Paris, France

**Keywords:** β-lactamase inhibitor, cyclic boronate acid β-lactamase inhibitor, β-lactam, *Mycobacterium abscessus*, xeruborbactam, tebipenem

## Abstract

Non-tuberculosis mycobacteria (NTM), particularly *Mycobacterium abscessus* subsp. *abscessus* (*M. abscessus*), are increasingly being recognized as etiological agents of NTM pulmonary disease. However, treatment options for *M. abscessus* are limited owing to their natural resistance to most antibiotics, including β-lactams. *M. abscessus* produces a class A β-lactamase, whose activity is inhibited by cyclic boronic acid β-lactamase inhibitors. We aimed to evaluate the *in vitro* effects of xeruborbactam, a cyclic boronic acid β-lactamase inhibitor, against *M. abscessus* when combined with five β-lactams (amoxicillin, tebipenem, cefdinir, cefuroxime, and cefoxitin). The drug susceptibilities of 43 *M*. *abscessus* clinical isolates obtained from 43 patients between August 2005 and May 2014 were tested. The MIC results for each β-lactam with or without 4 µg/mL xeruborbactam were examined. Xeruborbactam lowered the MIC_90_ values of tebipenem, amoxicillin, cefuroxime, and cefdinir by 5, ≥4, 3, and 3 dilutions, respectively. The MIC_90_ values of cefoxitin without xeruborbactam were 32 µg/mL and did not change upon the addition of xeruborbactam. The lowest MIC_90_ value was obtained for tebipenem with xeruborbactam. Almost all isolates had an MIC of 4 µg/mL; one isolate had an MIC of 2 µg/mL. With respect to the susceptibility to the same family drug, the number of susceptible isolates increased from 1/43 (2%) to 43/43 (100%) for tebipenem with xeruborbactam. Combining tebipenem and xeruborbactam could be considered an effective all-oral regimen that benefits outpatient treatment of *M. abscessus* pulmonary disease.

**IMPORTANCE:**

*Mycobacterium abscessus* subsp. *abscessus* (*M. abscessus*) disease is treated in two phases; injectable drugs for initial followed by others for continuation. There is a need to develop all-oral treatment methods for *M. abscessus* infection, especially in the continuation phase. However, treatment options for *M. abscessus* are limited owing to their natural resistance to most antibiotics. This is the first report to evaluate the in vitro effects of xeruborbactam, a cyclic boronic acid β-lactamase inhibitor capable of inhibiting the class A β-lactamase produced by *M. abscessus*, against 43 *M. abscessus* clinical isolates when combined with five β-lactam antibiotics. Xeruborbactam lowered the MIC90 values of tebipenem by five dilutions, and the number of susceptible isolates increased from 1/43 (2%) to 43/43 (100%). We showed that the tebipenem-xeruborbactam combination might be of interest to explore further as a potentially effective oral regimen for outpatient treatment of *M. abscessus* pulmonary disease.

## INTRODUCTION

Non-tuberculosis mycobacteria (NTM) are normally present in natural and living environments, and the prevalence of NTM pulmonary disease has been increasing globally (1). Among these, the *Mycobacterium abscessus* complex (MABC) stands out as an emerging pathogen. In Asia, including Japan, it is the second most prevalent pathogen after the *Mycobacterium avium-intracellulare* complex (1, 2). MABC comprises three subspecies, i.e., *M. abscessus* subsp. *abscessus* (*M. abscessus*), *M. abscessus* subsp. *massiliense* (*M. massiliense*), and *M. abscessus* subsp. *bolletii* (*M. bolletii*). MABCs are rapidly growing mycobacteria and cause invasive pulmonary infections, particularly in patients with structural lung disorders, such as cystic fibrosis and bronchiectasis (1).

MABC differs in susceptibility to the key drug, macrolide, based on subspecies. Though most *M. abscessus* and *M. bolletii* are macrolide-resistant, *M. massiliense* is macrolide-susceptible. Two primary mechanisms underlie their macrolide resistance. One mechanism involves inducible resistance owing to the erythromycin ribosomal resistance methylase, *erm*(41) (3). Partial truncation of the *erm*(41) observed in *M. massiliense* causes macrolide susceptibility. The T28C sequence variant (sequevar) observed in *M. abscessus* fails to function in the *erm*(41) gene and confers macrolide susceptibility. In both subspecies, as with other NTM, mutations in the 23S rRNA gene *rrl* cause high macrolide resistance, regardless of the *erm*(41) activity (4).

The current recommendations suggest a two-phase treatment approach for MABC pulmonary disease. The initial phase involves a combination of injectable antibiotics, followed by a continuation phase primarily using oral or inhaled antibiotics (5). Although multidrug therapy is recommended for MABC pulmonary disease, the treatment failure rate is high (6), and treatment options are limited owing to the natural resistance of MABC to many antibiotics. In the continuation phase, clofazimine and linezolid are the only two oral antibiotics recommended for use, and their long-term use is complicated because of adverse events (7, 8). Owing to the long-term use of antibiotics in the continuation phase, the development of novel and highly effective all-oral anti-MABC treatments is urgently needed.

MABC produces the class A β-lactamase, Bla_Mab_, which can hydrolyze penicillin, most cephalosporins, and carbapenems. Class A β-lactamases are not inhibited by traditional β-lactamase inhibitors, such as clavulanic acid, tazobactam, and sulbactam (9). In contrast, diazabicyclooctane β-lactamase and cyclic boronic acid β-lactamase inhibitors can inhibit Bla_Mab_ (10–16). Combinatorial treatment involving β-lactams and these novel β-lactamase inhibitors against MABC has predominantly been studied *in vitro*. Xeruborbactam is a cyclic boronic acid β-lactamase inhibitor with inhibitory activity against major members of classes A, B, C, and D β-lactamases. It is available for intravenous or oral administration and has completed phase 1 clinical trials (ClinicalTrials.gov identifiers NCT04380207 and NCT04578873). Therefore, we hypothesized that combining β-lactams and xeruborbactam might restore the susceptibility of MABC to β-lactams.

MABC has two distinct colony morphotypes, which exist as smooth and rough colonies. A recent study reported that patients with rough MABC morphotypes had worse clinical outcomes than those with smooth morphotypes (17).

This study aimed to evaluate the *in vitro* effects of five clinically available β-lactams (amoxicillin, tebipenem, cefdinir, cefuroxime, and cefoxitin) against *M. abscessus* when combined with xeruborbactam. Furthermore, a secondary objective was to study the differences in the effects of the two morphotypes.

## MATERIALS AND METHODS

### Bacterial strains

Forty-three *M. abscessus* isolates obtained primarily from airway specimens of 43 patients at Fukujuji Hospital, Japan Anti-Tuberculosis Association, between August 2005 and May 2014 were investigated. *M. abscessus* was cultured on 2% Ogawa medium (Kyokuto Pharmaceutical Industrial, Tokyo, Japan), an egg-based medium mainly used in Japan and some Asian countries instead of the Löwenstein-Jensen medium, at 30°C. Species and subspecies were identified based on multiplex PCR results and *rpoB* sequences according to previous reports (18, 19). The sequence of *erm*(41) was determined using the pyrosequencing method, as reported previously (20). All isolates were stored at −80°C at the Research Institute of Tuberculosis, Japan Anti-Tuberculosis Association, until use. As this study involved the use of only clinical isolates and no additional samples or personal information were obtained from the patients, ethics approval was not required.

### Antibiotics

Cefdinir (98% purity) and cefuroxime (100%) were obtained from Cayman Chemical (Ann Arbor, MI, USA), and amoxicillin (87.2%), tebipenem (100%), and cefoxitin (95.5%) were obtained from Sigma-Aldrich (St. Louis, MO, USA). (1R,2S)-Xeruborbactam (disodium; 95%) was obtained from Chem Scene LLC (Monmouth Junction, NJ, USA), and as per the manufacturer’s instructions, it is soluble in dimethyl sulfoxide; there are no data available about the water solubility of the compound. We dissolved xeruborbactam and individual β-lactams in cation-adjusted Mueller-Hinton broth (CAMHB; pH 7.4) and stored the solutions at −80°C until use. When measuring the MIC values of individual β-lactams, a two-fold dilution series of the β-lactam stock solution was prepared using CAMHB. However, when measuring the MIC values of individual combinations of β-lactams and xeruborbactam, a two-fold dilution series of the β-lactam stock solution was prepared using CAMHB containing xeruborbactam.

Given this study’s aim to explore an oral MABC regimen suitable for the continuation phase, we selected the commercially available oral β-lactams, amoxicillin, tebipenem, cefdinir, and cefuroxime, which were previously reported to be effective when combined with novel β-lactamase inhibitors (10, 11, 13–16, 21). Cefoxitin was already included as the treatment option in the ATS/ERS/ESCMID/IDSA clinical practice guidelines (5) and was used as a reference in this study. The MIC of xeruborbactam was not measured because novel β-lactamase inhibitors were previously demonstrated to lack antibacterial activity (13, 14).

### Antimicrobial susceptibility test

The MIC for each isolate was determined using the broth microdilution method with 96-well microtiter plates (Thermo Fisher Scientific, Waltham, MA, USA). The culture medium used was CAMHB (pH 7.4), as per the guidelines outlined in Clinical and Laboratory Standards Institute M24, third edition (22). Antibiotic solutions were prepared with a twofold dilution. The final drug concentrations were as follows: amoxicillin, tebipenem, cefdinir, and cefuroxime (0.25–256 µg/mL); cefoxitin (0.125–128 µg/mL). Xeruborbactam was used at a final concentration of 4 µg/mL, a level that can be easily attained in human plasma (23–25). The half-life of xeruborbactam is estimated to be approximately 30 h (24, 25), while that of avibactam is approximately 2 h (26). Although some β-lactams, such as imipenem, are unstable during long incubation time, a phenomenon that could potentially result in artificially high MIC values, avibactam is reported to be stable during incubation (14, 27). Therefore, we have considered that xeruborbactam was probably stable during incubation.

Bacterial colonies were transferred to test tubes with 5 mm diameter glass beads. Sterile distilled water was subsequently added, and the tubes were agitated using a vortex mixer to disperse any aggregated bacteria and ensure even suspension. Next, the bacterial suspension was resuspended in sterile distilled water and adjusted to a McFarland 0.5 standard by measuring absorbance at an optical density of 530 nm (OD_530_). The final inoculum suspensions, each having a concentration of 5 × 10^5^ CFU/mL, were prepared by transferring 90 µL of the 0.5 McFarland suspensions to 9 mL CAMHB. Subsequently, 50 µL of this final inoculum suspension was added to each well of a microtiter plate containing 50 µL of CAMHB with the dissolved antimicrobial agent, resulting in a final bacterial density of approximately 5 × 10^4^ CFU in each well. Plates were incubated at 30°C under aerobic conditions. When the control strain grew sufficiently (on days 3–5), the MIC values for individual β-lactams were assessed. If growth remained insufficient on day 5, incubation was extended to 14 days until sufficient growth was achieved.

The colony morphotype was decided based on appearance with the naked eye and sense of touch with the inoculating loop; the smooth morphotypes are characterized by shiny, smooth, and wet colonies, and the rough morphotypes are characterized by larger, markedly rugged, waxier, and dry colonies. The 96-well microtiter plates and colonies growing on 2% Ogawa medium were observed by more than two persons, and the final MIC values and colony morphotypes were determined by consensus.

Quality control (QC) was performed using *Mycobacterium peregrinum* ATCC 700686. The QC range was applied as recommended in CLSI M24S (28). Additionally, *M. abscessus* ATCC 19977 was used as an internal QC strain.

MIC_50_ and MIC_90_ were defined as the antibiotic concentrations preventing the growth of 50% and 90% of the isolates, respectively. The method for determining the MIC_50_ and MIC_90_ involved ordering the MIC values in ascending order and visually confirming which concentration corresponded to 50% and 90% inhibition, respectively.

Among the drugs tested in this study, cefoxitin was the only drug with established breakpoints for rapidly growing mycobacteria according to the CLSI guidelines (28). For the remaining drugs, we referred to the susceptibility of the same family drugs, imipenem and meropenem (MIC ≤ 4 µg/mL is susceptible), in addition to that of cefoxitin (MIC ≤ 16 µg/mL is susceptible).

### Statistical analysis

Statistical analysis was performed using the EZR version 1.62 (Saitama Medical Center, Jichi Medical University, Japan). As the data presented in this study were not normally distributed, nonparametric methods were used for analysis. Continuous variables for paired samples were compared using the Wilcoxon signed-rank test. All *P*-values were two-sided, and statistical significance was set at *P* < 0.05.

## RESULTS

### Identification of subspecies, erm(41) sequences, and colony morphotypes

All 43 isolates were identified as *M. abscessus*. Among these 43 *M*. *abscessus* isolates, 41 (95%) were associated with the T28 sequevar, while 2 (5%) were associated with the T28C sequevars. Among the tested isolates, 20 (18 clinical isolates and 2 QC strains) exhibited the rough morphotype, and 25 exhibited the smooth morphotype. None of the colonies were of indeterminate morphotype.

### Evaluation of quality control

QC was performed using *M. peregrinum* ATCC 700686. The MIC of cefoxitin in QC was 8 µg/mL (Table 1), within the QC range (4–32 µg/mL) mentioned in CLSI M24S (28).

**TABLE 1:** MIC values of β-lactam antibiotics with or without 4 µg/mL xeruborbactam against *M. peregrinum* ATCC700686 and *M. abscessus* subsp. *abscessus* ATCC 19977 in CAMHB

Strain	β-Lactam antibiotic	MIC (µg/mL)	
		Alone	With xeruborbactam
*M. peregrinum* ATCC700686	Amoxicillin	256	16
	Tebipenem	2	4
	Cefdinir	128	>256
	Cefuroxime	>256	>256
	Cefoxitin	8	8
*M. abscessus* ATCC19977	Amoxicillin	>256	8
	Tebipenem	64	4
	Cefdinir	128	16
	Cefuroxime	256	32
	Cefoxitin	16	32

### Activity of β-lactams with or without xeruborbactam against the 43 clinical isolates

**Fig 1:**
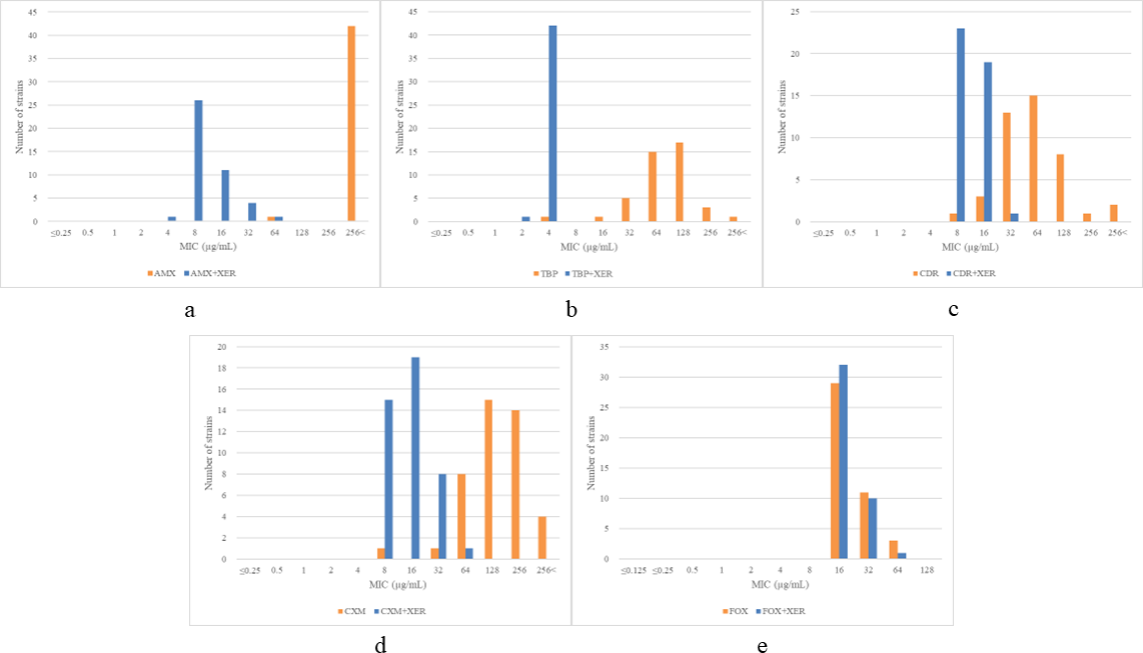
MIC distributions of β-lactam antibiotics with or without 4 µg/mL xeruborbactam against the 43 *M*. *abscessus* subsp. *abscessus* clinical strains. (a) AMX; (b) TBP; (c) CDR; (d) CXM; (e) FOX. AMX, amoxicillin; CDR, cefdinir; CXM, cefuroxime; FOX, cefoxitin; TBP, tebipenem; XER, xeruborbactam.

The MIC values for individual β-lactams with or without 4 µg/mL xeruborbactam against the 43 *M*. *abscessus* clinical isolates were evaluated. Results showed that the MIC values tended to be higher for the rough morphotypes than for the smooth morphotypes (by one dilution; Table 2). For one of the clinical isolates (No. 7), adequate growth was not observed within the initial 5 days; therefore, we continued the incubation and determined the MIC value on day 10. The MIC distributions of individual β-lactams with or without 4 µg/mL xeruborbactam are shown in Fig. 1, and their MIC range, MIC_50_, and MIC_90_ values are presented in Table 2. The MIC_90_ value for individual β-lactams (except for cefoxitin) exceeded 128 µg/mL, which was not expected for antimicrobial activity. The MIC_90_ value of cefoxitin was slightly low at 32 µg/mL compared to that of other β-lactams, corresponding to intermediate susceptibility when referring to CLSI M24S (28). As with the *M. abscessus* ATCC 19977 strain, xeruborbactam lowered the MIC values of all β-lactams, except for cefoxitin, against the 43 clinical isolates. The MIC range shifted significantly to lower values in the following order: tebipenem > amoxicillin > cefuroxime > cefdinir. The lowest MIC_90_ value was obtained from tebipenem with xeruborbactam; 1/43 isolates (2%) had an MIC of 2 µg/mL, and 42/43 isolates (98%) had an MIC of 4 µg/mL. The Wilcoxon signed-rank test indicated significant differences for amoxicillin (*P* < 0.001), tebipenem (*P* < 0.001), cefuroxime (*P* < 0.001), and cefdinir (*P* < 0.001) but not for cefoxitin (*P* = 0.124). The MIC_50_ and MIC_90_ values of cefoxitin without xeruborbactam were 16 µg/mL and 32 µg/mL, respectively, and they did not change upon the addition of xeruborbactam.

**TABLE 2:** MIC values of β-lactam antibiotics with or without 4 µg/mL xeruborbactam against 43 *M*. *abscessus* subsp. *abscessus* clinical strains divided according to colony morphotype in CAMHB^*a*^

Antibiotic(s)	MIC (µg/mL)						
	Range	MIC_50_			MIC_90_		
		All	Smooth	Rough	All	Smooth	Rough
AMX	64–>256	>256	>256	>256	>256	>256	>256
AMX with XER	4–64	8	8	8	32	16	32
TBP	4–>256	64	64	128	128	128	128
TBP with XER	2–4	4	4	4	4	4	4
CDR	8–>256	64	64	64	128	128	128
CDR with XER	8–32	8	8	16	16	16	16
CXM	8–>256	128	128	256	256	>256	256
CXM with XER	8–64	16	8	16	32	16	32
FOX	16–64	16	16	32	32	64	32
FOX with XER	16–64	16	16	32	32	32	32

^*a*^ AMX, amoxicillin; CDR, cefdinir; CXM, cefuroxime; FOX, cefoxitin; TBP, tebipenem; XER, xeruborbactam.

## DISCUSSION

This study demonstrated that xeruborbactam restored the activity of certain β-lactams against *M. abscessus*, which is most notably seen for tebipenem. Xeruborbactam lowered the MIC_90_ values of tebipenem, amoxicillin, cefuroxime, and cefdinir by 5, ≥4, 3, and 3 dilutions, respectively.

Diazabicyclooctane β-lactamase (avibactam [10–12], relebactam [13, 14], durlobactam [15], zidebactam [16], and nacubactam [13, 16]) and cyclic boronic acid β-lactamase (vaborbactam [14]) inhibitors reportedly inhibit Bla_Mab_. The use of combination therapy involving β-lactams along with novel β-lactamase inhibitors against MABC has primarily been investigated *in vitro*. Only two *in vivo* studies (29, 30) and one case report on using imipenem-relebactam for *M. abscessus* skin and soft tissue infection are available (31). To our knowledge, this was the first described case of the successful treatment of an *M. abscessus* infection using a novel β-lactamase inhibitor.

Previous reports indicated that novel β-lactamase inhibitors usually lowered the MIC values of tebipenem, cefuroxime, and cefdinir by 1–5, 2–4 or more, and 1–2 dilutions, respectively (10, 11, 13–16, 21). The decrease in the MIC values of amoxicillin varied according to the report, sometimes decreasing by approximately three dilutions or not at all. In this study, the MIC values of tebipenem, cefuroxime, cefdinir, and amoxicillin were reduced when these drugs were used in combination with xeruborbactam, and tebipenem demonstrated the most significant reduction, which were predictable results. In Fig. 1a and b, a well-defined Gaussian distribution is observed for tebipenem. Interestingly, when xeruborbactam was added, all MIC values converged around 4 µg/mL. Kaushik et al. (12) suggested that this concentration might represent a lower limit for the combination of carbapenem and avibactam, potentially explaining the observed convergence of MIC values.

Among the drugs used in this study, only cefoxitin had established breakpoints for MABC as per the CLSI guidelines (28). Therefore, to circumvent this issue, the susceptibilities of the same family drugs, imipenem and meropenem (MIC ≤ 4 µg/mL is susceptible), in addition to those of cefoxitin (MIC ≤ 16 µg/ml is susceptible) were used as reference. In such cases, comparison of individual MICs with peak plasma concentration, half-life, and plasma protein binding may be useful clinically (tebipenem: peak plasma concentration 14.1 µg/mL, half-life 1 h, and plasma protein binding 50% [32, 33]; imipenem: peak plasma concentration 30–35 µg/mL, half-life 1 h, and plasma protein binding 20% [34]; meropenem: peak plasma concentration 26 µg/mL, half-life 1 h, and plasma protein binding 2% [34]; cefuroxime: peak plasma concentration 2.1–13.6 µg/mL, half-life 2.2–3 h, and plasma protein binding 33%–50% [35]; cefdinir: peak plasma concentration 1.6–2.87 µg/mL, 1.5–1.7 h, and plasma protein binding 60%–73% [36]; cefoxitin: peak plasma concentration 42 µg/mL, 49 min, and plasma protein binding 50% [37]). With reference to the above susceptibilities, the number of susceptible isolates increased from 2.3% (1 of 43) to 79.1% (34 of 43) for cefuroxime, from 9.3% (4 of 43) to 97.7% (42 of 43) for cefdinir, and from 2.3% (1 of 43) to 100% (43 of 43) for tebipenem with xeruborbactam. All isolates became susceptible when tebipenem was used with xeruborbactam. A systematic review of the combination therapy with carbapenems and novel β-lactamase inhibitors showed that the combination of tebipenem with avibactam against MABC was effective (21). Previous studies on multiple MABC clinical isolates reported that adding avibactam to tebipenem reduced the MIC_50_ from 256 to 16 µg/mL (38). Although our study included a smaller number of isolates, the addition of xeruborbactam to tebipenem also led to a significant reduction in MIC_50_, from 64 to 4 µg/mL. These findings suggest that the combination of tebipenem and xeruborbactam could be a promising addition to the treatment options for MABC infections.

The combinations ceftazidime-avibactam, sulbactam-durlobactam, imipenem-relebactam, and meropenem-vaborbactam are commercially available in the United States. However, ceftazidime is not active against MABC, even in combination with avibactam. Additionally, it is not effective against *M. abscessus* in which the gene encoding Bla_Mab_ is deleted (9, 10, 39). Although sulbactam inhibits penicillin-binding protein, it is potent at only high concentrations (39, 40). Thus, the combination of β-lactam with ceftazidime-avibactam or sulbactam-durlobactam is needed to ensure efficient Bla_Mab_ inhibition. However, this introduces the risk of ceftazidime- or sulbactam-related adverse events. Using ceftazidime-avibactam or sulbactam-durlobactam for MABC therapy would be inappropriate. In contrast, imipenem-relebactam and meropenem-vaborbactam may be active against MABC (14); nevertheless, both drugs need intravenous administration, and using them as outpatient treatment in the continuation phase is complicated.

Xeruborbactam is being developed as an injectable form with meropenem and an oral form with ceftibuten (41). Although it is unknown whether these combinations are effective against MABC, these can be administered both intravenously and orally and would likely be suitable for treating MABC, particularly as an all-oral regimen for outpatient treatment in the continuation phase. Furthermore, the combination of dual β-lactams and novel β-lactamase inhibitors has recently been reported to be more effective against MABC (27). Evaluation of the combined effects of meropenem-xeruborbactam or ceftibuten-xeruborbactam with other β-lactams for real-world clinical use is essential.

The primary adverse events of tebipenem, cefuroxime, and cefdinir are diarrhea, nausea, vomiting, and headache; however, most of them are mild or moderate in severity and non-treatment-limiting (35, 36, 42). Xeruborbactam has completed phase 1 clinical trials (NCT04380207, NCT04578873) in healthy adults administered through the intravenous or oral route. In these reports, xeruborbactam was safe and well tolerated at exposures that exceeded non-clinical PK-PD targets (24, 25). Because combining these β-lactams and xeruborbactam may cause unexpected adverse events or increase their incidence rates, further investigation is needed. Considering the incidence rate of the adverse events of currently recommended therapies, studying this combination is crucial.

As previously observed with avibactam, relebactam, nacubactam, zidebactam, and vaborbactam (10, 14, 16), the MIC value of cefoxitin was not affected by the presence of xeruborbactam (MIC_50_ of 16 µg/mL and MIC_90_ of 32 µg/mL with or without xeruborbactam). Soroka et al. (9) and Dubée et al. (43) reported that cefoxitin was slowly hydrolyzed by Bla_Mab_, revealing its stability in the presence of Bla_Mab_.

We analyzed the differences in the MIC values by colony morphotype. The MIC values tended to be higher for rough morphotypes than for smooth morphotypes by one dilution. The difference between the smooth and rough morphotypes is based on the glycopeptidolipids (GPLs) in the mycobacterial cell wall; smooth morphotypes possess GPL, whereas rough morphotypes do not. GPL conveys hydrophilicity, and the absence of GPL increases bacterial hydrophobicity (44). All antibiotics used in this study were hydrophilic, which could account for the low susceptibility of the rough morphotypes. Furthermore, the absence of GPL facilitates bacterial aggregation, clumping, and cording (44). This may have prevented the drug from reaching the bacteria. The same results were reported in previous studies (13, 45). Although there are *in vitro* studies, the recent retrospective multicenter cohort study focused on *in vitro* investigations revealed that patients infected with rough MABC colony morphotypes experienced poorer clinical outcomes, including cavitary pulmonary disease and a higher frequency of cough, compared to those with smooth isolates (17). Consequently, when culturing MABC, it may be prudent to consider not only MIC values but also the colony morphotypes.

This study has some limitations. First, because the clinical isolates used in this study were obtained from a single hospital, the number of isolates was relatively small and may differ from those in other locations. Second, only *M. abscessus* isolates were evaluated in this study. A previous study showed that the proportion of patients with sustained sputum conversion rate without relapse was 23% for *M. abscessus* and 84% for *M. massiliense*, owing to differences in macrolide susceptibility (6, 46). Furthermore, obtaining *M. bollettii* strains was challenging because *M. bollettii* pulmonary disease cases are rare in Japan (47). This is why we focused on *M. abscessus* strains in this study. Third, the results of *in vitro* studies might not be applicable to clinical cases because MIC values reflect results in the predominant strains and likely do not reflect results in the subpopulation. However, we confirmed the repeatability and reproducibility using each β-lactam alone on the QC strain. Further *in vitro* and *in vivo* studies are needed to evaluate whether combining xeruborbactam with cefuroxime, cefdinir, and tebipenem is effective for treating other clinical strains, the extent of their bactericidal action and therapeutic efficacy, and the incidence of adverse events.

To the best of our knowledge, this study is the first to evaluate the effect of combining xeruborbactam with β-lactams against *M. abscessus*, and the findings of this study are useful because of the need to develop novel and highly effective all-oral anti-MABC regimens. Xeruborbactam lowered the MIC_90_ of tebipenem, amoxicillin, cefuroxime, and cefdinir by 5, ≥4, 3, and 3 dilutions, respectively. The lowest MIC_90_ value was obtained from tebipenem with xeruborbactam, indicating that all isolates possibly became susceptible when using this combination. The combination of tebipenem and xeruborbactam could be considered effective for total oral regimens used in the outpatient treatment of *M. abscessus* pulmonary disease.

## Supplementary Material

Reviewer comments

## SUPPLEMENTAL MATERIAL

OPEN PEER REVIEW reviewer-comments.pdf An accounting of the reviewer comments and feedback.

ASM does not own the copyrights to Supplemental Material that may be linked to, or accessed through, an article. The authors have granted ASM a non-exclusive, world-wide license to publish the Supplemental Material files. Please contact the corresponding author directly for reuse.

## References

[1] Prevots DR, Marshall JE, Wagner D, Morimoto K. 2023. Global epidemiology of nontuberculous mycobacterial pulmonary disease: a review. Clin Chest Med 44:675–721. doi:10.1016/j.ccm.2023.08.012 37890910 PMC10625169

[2] Morimoto K, Hasegawa N, Izumi K, Namkoong H, Uchimura K, Yoshiyama T, Hoshino Y, Kurashima A, Sokunaga J, Shibuya S, Shimojima M, Ato M, Mitarai S. 2017. A laboratory-based analysis of nontuberculous mycobacterial lung disease in Japan from 2012 to 2013. Ann Am Thorac Soc 14:49–56. doi:10.1513/AnnalsATS.201607-573OC 27788025

[3] Bastian S, Veziris N, Roux AL, Brossier F, Gaillard JL, Jarlier V, Cambau E. 2011. Assessment of clarithromycin susceptibility in strains belonging to the Mycobacterium abscessus group by erm(41) and rrl sequencing. Antimicrob Agents Chemother 55:775–781. doi:10.1128/AAC.00861-10 21135185 PMC3028756

[4] Wallace RJ, Meier A, Brown BA, Zhang Y, Sander P, Onyi GO, Böttger EC. 1996. Genetic basis for clarithromycin resistance among isolates of Mycobacterium chelonae and Mycobacterium abscessus. Antimicrob Agents Chemother 40:1676–1681. doi:10.1128/AAC.40.7.1676 8807061 PMC163394

[5] Daley CL, Iaccarino JM, Lange C, Cambau E, Wallace RJ, Andrejak C, Böttger EC, Brozek J, Griffith DE, Guglielmetti L, Huitt GA, Knight SL, Leitman P, Marras TK, Olivier KN, Santin M, Stout JE, Tortoli E, van Ingen J, Wagner D, Winthrop KL. 2020. Treatment of nontuberculous mycobacterial pulmonary disease: an official ATS/ERS/ESCMID/IDSA clinical practice guideline. Clin Infect Dis 71:905–913. doi:10.1093/cid/ciaa241 32797222 PMC7768745

[6] Pasipanodya JG, Ogbonna D, Ferro BE, Magombedze G, Srivastava S, Deshpande D, Gumbo T. 2017. Systematic review and meta-analyses of the effect of chemotherapy on pulmonary Mycobacterium abscessus outcomes and disease recurrence. Antimicrob Agents Chemother 61:e01206-17. doi:10.1128/AAC.01206-17 28807911 PMC5655093

[7] Yang B, Jhun BW, Moon SM, Lee H, Park HY, Jeon K, Kim DH, Kim SY, Shin SJ, Daley CL, Koh WJ. 2017. Clofazimine-containing regimen for the treatment of Mycobacterium abscessus lung disease. Antimicrob Agents Chemother 61:e02052-16. doi:10.1128/AAC.02052-16 28348153 PMC5444135

[8] Winthrop KL, Ku JH, Marras TK, Griffith DE, Daley CL, Olivier KN, Aksamit TR, Varley CD, Mackey K, Prevots DR. 2015. The tolerability of Linezolid in the treatment of nontuberculous mycobacterial disease. Eur Respir J 45:1177–1179. doi:10.1183/09031936.00169114 25614169 PMC6660918

[9] Soroka D, Dubée V, Soulier-Escrihuela O, Cuinet G, Hugonnet JE, Gutmann L, Mainardi JL, Arthur M. 2014. Characterization of broad-spectrum Mycobacterium abscessus class A β-lactamase. J Antimicrob Chemother 69:691–696. doi:10.1093/jac/dkt410 24132992

[10] Dubée V, Bernut A, Cortes M, Lesne T, Dorchene D, Lefebvre AL, Hugonnet JE, Gutmann L, Mainardi JL, Herrmann JL, Gaillard JL, Kremer L, Arthur M. 2015. β-lactamase inhibition by avibactam in Mycobacterium abscessus. J Antimicrob Chemother 70:1051–1058. doi:10.1093/jac/dku510 25525201

[11] Story-Roller E, Maggioncalda EC, Lamichhane G. 2019. Select β-lactam combinations exhibit synergy against Mycobacterium abscessus in vitro. Antimicrob Agents Chemother 63:e02613-18. doi:10.1128/AAC.02613-18 30745389 PMC6437493

[12] Kaushik A, Gupta C, Fisher S, Story-Roller E, Galanis C, Parrish N, Lamichhane G. 2017. Combinations of avibactam and carbapenems exhibit enhanced potencies against drug-resistant Mycobacterium abscessus. Future Microbiol 12:473–480. doi:10.2217/fmb-2016-0234 28326811 PMC5618940

[13] Misawa K, Nishimura T, Kashimura S, Enoki Y, Taguchi K, Uno S, Uwamino Y, Matsumoto K, Hasegawa N. 2022. In vitro effects of diazabicyclooctane β-lactamase inhibitors relebactam and nacubactam against three subspecies of Mycobacterium abscessus complex. Int J Antimicrob Agents 60:106669. doi:10.1016/j.ijantimicag.2022.106669 36064079

[14] Kaushik A, Ammerman NC, Lee J, Martins O, Kreiswirth BN, Lamichhane G, Parrish NM, Nuermberger EL. 2019. In vitro activity of the new β-lactamase inhibitors relebactam and vaborbactam in combination with β-lactams against Mycobacterium abscessus complex clinical isolates. Antimicrob Agents Chemother 63:e02623-18. doi:10.1128/AAC.02623-18 30642943 PMC6395916

[15] Dousa KM, Nguyen DC, Kurz SG, Taracila MA, Bethel CR, Schinabeck W, Kreiswirth BN, Brown ST, Boom WH, Hotchkiss RS, Remy KE, Jacono FJ, Daley CL, Holland SM, Miller AA, Bonomo RA. 2022. Inhibiting Mycobacterium abscessus cell wall synthesis: using a novel diazabicyclooctane β-Lactamase inhibitor to augment β-lactam action. mBio 13:e0352921. doi:10.1128/mbio.03529-21 35073757 PMC8787486

[16] Kaushik A, Ammerman NC, Parrish NM, Nuermberger EL. 2019. New β-lactamase inhibitors nacubactam and zidebactam improve the in vitro activity of β-lactam antibiotics against Mycobacterium abscessus complex clinical isolates. Antimicrob Agents Chemother 63:e00733-19. doi:10.1128/AAC.00733-19 31209013 PMC6709484

[17] Hedin W, Fröberg G, Fredman K, Chryssanthou E, Selmeryd I, Gillman A, Orsini L, Runold M, Jönsson B, Schön T, Davies Forsman L. 2023. A rough colony morphology of Mycobacterium abscessus is associated with cavitary pulmonary disease and poor clinical outcome. J Infect Dis 227:820–827. doi:10.1093/infdis/jiad007 36637124 PMC10043986

[18] Nakanaga K, Sekizuka T, Fukano H, Sakakibara Y, Takeuchi F, Wada S, Ishii N, Makino M, Kuroda M, Hoshino Y. 2014. Discrimination of Mycobacterium abscessus subsp. massiliense from Mycobacterium abscessus subsp. abscessus in clinical isolates by multiplex PCR. J Clin Microbiol 52:251–259. doi:10.1128/JCM.01327-13 24197885 PMC3911466

[19] Adékambi T, Berger P, Raoult D, Drancourt M. 2006. rpoB gene sequence-based characterization of emerging non-tuberculous mycobacteria with descriptions of Mycobacterium bolletii sp. nov., Mycobacterium phocaicum sp. nov. and Mycobacterium aubagnense sp. nov. Int J Syst Evol Microbiol 56:133–143. doi:10.1099/ijs.0.63969-0 16403878

[20] Yoshida S, Tsuyuguchi K, Suzuki K, Tomita M, Okada M, Shimada R, Hayashi S. 2014. Rapid identification of strains belonging to the Mycobacterium abscessus group through erm(41) gene pyrosequencing. Diagn Microbiol Infect Dis 79:331–336. doi:10.1016/j.diagmicrobio.2014.04.001 24809859

[21] Andersson V, Fröberg G, Dahl VN, Chryssanthou E, Giske C, Schön T, Forsman LD. 2023. The in vitro activity of carbapenems alone and in combination with β-lactamase inhibitors against difficult-to-treat mycobacteria; Mycobacterium tuberculosis, Mycobacterium abscessus, and Mycobacterium avium complex: a systematic review. Int J Mycobacteriol 12:211–225. doi:10.4103/ijmy.ijmy_131_23 37721224

[22] Woods GL, Wengenack NL, Lin G, Brown-Elliott BA, Cirillo DM, Conville PS, Desmond EP, Scott B, Killian B, Parrish NM, Pfeltz R, Richter E, Turnidge JD. 2018. Susceptibility testing of *Mycobacteria, Nocardia* spp., and other aerobic actinomycetes. In CLSI standard document M24, 3rd ed. Clinical and Laboratory Standards Institute, Wayne, PA, USA. 31339680

[23] Hecker SJ, Reddy KR, Lomovskaya O, Griffith DC, Rubio-Aparicio D, Nelson K, Tsivkovski R, Sun D, Sabet M, Tarazi Z, Parkinson J, Totrov M, Boyer SH, Glinka TW, Pemberton OA, Chen Y, Dudley MN. 2020. Discovery of cyclic boronic acid QPX7728, an ultrabroad-spectrum inhibitor of serine and metallo-β-lactamases. J Med Chem 63:7491–7507. doi:10.1021/acs.jmedchem.9b01976 32150407

[24] Griffith D, Roberts J, Wallis S, Hernandez-Mitre MP, Morgan E, Dudley M, Loutit J. 2022. A phase 1 study of the single-dose safety, tolerability, and pharmacokinetics of the beta-lactamase inhibitor xeruborbactam administered as the isobutyryloxymethyl oral prodrug to healthy adult subjects. Open Forum Infect Dis 9. doi:10.1093/ofid/ofac492.296

[25] Griffith D, Roberts J, Wallis S, Hernandez-Mitre MP, Morgan E, Gehrke S, Dudley M, Loutit J. 2022. A phase 1 study of the safety tolerability, and pharmacokinetics of multiple doses of the beta-lactamase inhibitor xeruborbactam alone and in combination meropenem in healthy adult subjects. Open Forum Infect Dis 9. doi:10.1093/ofid/ofac492.294

[26] Zhanel GG, Lawson CD, Adam H, Schweizer F, Zelenitsky S, Lagacé-Wiens PRS, Denisuik A, Rubinstein E, Gin AS, Hoban DJ, Lynch JP 3rd, Karlowsky JA. 2013. Ceftazidime-avibactam: a novel cephalosporin/β-lactamase inhibitor combination. Drugs 73:159–177. doi:10.1007/s40265-013-0013-7 23371303

[27] Negatu DA, Zimmerman MD, Dartois V, Dick T. 2022. Strongly bactericidal all-oral β-lactam combinations for the treatment of Mycobacterium abscessus lung disease. Antimicrob Agents Chemother 66:e0079022. doi:10.1128/aac.00790-22 36047786 PMC9487536

[28] Nicole MP, Nancy LW, Barker A, Brown-Elliott BA, Cirillo DM, Harrington S, Khare R, Killian SB, Pfeltz R, Richter E, Rowlinson M-C, Zelazny AM. 2023. Performance standards for susceptibility testing of Mycobacteria, Nocardia spp., and other aerobic actinomycetes, 2nd ed, CLSI supplement M24S. Clinical and Laboratory Standards Institute, Wayne, PA, USA. 31339680

[29] Lefebvre AL, Le Moigne V, Bernut A, Veckerlé C, Compain F, Herrmann JL, Kremer L, Arthur M, Mainardi JL. 2017. Inhibition of the β-Lactamase Bla_Mab_ by avibactam improves the in vitro and in vivo efficacy of imipenem against Mycobacterium abscessus. Antimicrob Agents Chemother 61. doi:10.1128/AAC.02440-16 PMC5365697 28096155

[30] Negatu DA, González Del Río R, Cacho-Izquierdo M, Barros-Aguirre D, Lelievre J, Rullas J, Casado P, Ganapathy US, Zimmerman MD, Gengenbacher M, Dartois V, Dick T. 2023. Activity of oral tebipenem-avibactam in a mouse model of Mycobacterium abscessus lung infection. Antimicrob Agents Chemother 67:e0145922. doi:10.1128/aac.01459-22 36688684 PMC9933631

[31] Beech AJ, Weinberg SE, Mortimer AE, Lynch F, Bedford J, Calisti G. 2023. Mycobacterium abscessus skin and soft tissue infection following autologous fat grafting in Kurdistan treated with an antibiotic combination including imipenem-relebactam and rifabutin. J Clin Tuberc Other Mycobact Dis 32:100381. doi:10.1016/j.jctube.2023.100381 37323244 PMC10267594

[32] Patel G, Rodvold KA, Gupta VK, Bruss J, Gasink L, Bajraktari F, Lei Y, Jain A, Srivastava P, Talley AK. 2022. Pharmacokinetics of oral tebipenem pivoxil hydrobromide in subjects with various degrees of renal impairment. Antimicrob Agents Chemother 66:e0240721. doi:10.1128/aac.02407-21 35420493 PMC9112917

[33] Abouelhassan Y, Fratoni AJ, Shepard AK, Nicolau DP, Asempa TE. 2022. Pharmacokinetics and soft-tissue distribution of tebipenem pivoxil hydrobromide using microdialysis: a study in healthy subjects and patients with diabetic foot infections. J Antimicrob Chemother 78:296–301. doi:10.1093/jac/dkac399 36424364

[34] Zhanel GG, Wiebe R, Dilay L, Thomson K, Rubinstein E, Hoban DJ, Noreddin AM, Karlowsky JA. 2007. Comparative review of the carbapenems. Drugs 67:1027–1052. doi:10.2165/00003495-200767070-00006 17488146

[35] Scott LJ, Ormrod D, Goa KL. 2001. Cefuroxime axetil: an updated review of its use in the management of bacterial infections. Drugs 61:1455–1500. doi:10.2165/00003495-200161100-00008 11558834

[36] Perry CM, Scott LJ. 2004. Cefdinir: a review of its use in the management of mild-to-moderate bacterial infections. Drugs 64:1433–1464. doi:10.2165/00003495-200464130-00004 15212560

[37] Carver PL, Nightingale CH, Quintiliani R. 1989. Pharmacokinetics and pharmacodynamics of total and unbound cefoxitin and cefotetan in healthy volunteers. J Antimicrob Chemother 23:99–106. doi:10.1093/jac/23.1.99 2745258

[38] Fröberg G, Ahmed A, Chryssanthou E, Davies Forsman L. 2023. The in vitro effect of new combinations of carbapenem-β-lactamase inhibitors for Mycobacterium abscessus. Antimicrob Agents Chemother doi:10.1128/aac.00528-23:e0052823. PMC10583658 37671880

[39] Rominski A, Schulthess B, Müller DM, Keller PM, Sander P. 2017. Effect of β-lactamase production and β-lactam instability on MIC testing results for Mycobacterium abscessus. J Antimicrob Chemother 72:3070–3078. doi:10.1093/jac/dkx284 28961987

[40] Sayed ARM, Shah NR, Basso KB, Kamat M, Jiao Y, Moya B, Sutaria DS, Lang Y, Tao X, Liu W, Shin E, Zhou J, Werkman C, Louie A, Drusano GL, Bulitta JB. 2020. First penicillin-binding protein occupancy patterns for 15 β-lactams and β-lactamase inhibitors in Mycobacterium abscessus. Antimicrob Agents Chemother 65:e01956-20. doi:10.1128/AAC.01956-20 33106266 PMC7927833

[41] Veeraraghavan B, Bakthavatchalam YD, Sahni RD. 2021. Oral antibiotics in clinical development for community-acquired urinary tract infections. Infect Dis Ther 10:1815–1835. doi:10.1007/s40121-021-00509-4 34357517 PMC8572892

[42] Eckburg PB, Muir L, Critchley IA, Walpole S, Kwak H, Phelan AM, Moore G, Jain A, Keutzer T, Dane A, Melnick D, Talley AK. 2022. Oral tebipenem pivoxil hydrobromide in complicated urinary tract infection. N Engl J Med 386:1327–1338. doi:10.1056/NEJMoa2105462 35388666

[43] Dubée V, Soroka D, Cortes M, Lefebvre AL, Gutmann L, Hugonnet JE, Arthur M, Mainardi JL. 2015. Impact of β-lactamase inhibition on the activity of ceftaroline against Mycobacterium tuberculosis and Mycobacterium abscessus. Antimicrob Agents Chemother 59:2938–2941. doi:10.1128/AAC.05080-14 25733512 PMC4394810

[44] Johansen MD, Herrmann JL, Kremer L. 2020. Non-tuberculous mycobacteria and the rise of Mycobacterium abscessus. Nat Rev Microbiol 18:392–407. doi:10.1038/s41579-020-0331-1 32086501

[45] Fujiwara K, Aono A, Asami T, Morimoto K, Kamada K, Morishige Y, Igarashi Y, Chikamatsu K, Murase Y, Yamada H, Takaki A, Mitarai S. 2023. In vitro synergistic effects of omadacycline with other antimicrobial agents against Mycobacterium abscessus. Antimicrob Agents Chemother 67:e0157922. doi:10.1128/aac.01579-22 37154742 PMC10269086

[46] Yoshida S, Tsuyuguchi K, Suzuki K, Tomita M, Okada M, Hayashi S, Iwamoto T, Saito H. 2013. Further isolation of Mycobacterium abscessus subsp. abscessus and subsp. bolletii in different regions of Japan and susceptibility of these isolates to antimicrobial agents. Int J Antimicrob Agents 42:226–231. doi:10.1016/j.ijantimicag.2013.04.029 23850022

[47] Yoshida M, Chien JY, Morimoto K, Kinjo T, Aono A, Murase Y, Fujiwara K, Morishige Y, Nagano H, Jou R, Hasegawa N, Ato M, Hoshino Y, Hsueh PR, Mitarai S. 2022. Molecular epidemiological characteristics of Mycobacterium abscessus complex derived from non-cystic fibrosis patients in Japan and Taiwan. Microbiol Spectr 10:e0057122. doi:10.1128/spectrum.00571-22 35446117 PMC9248903

